# Smart textiles using fluid-driven artificial muscle fibers

**DOI:** 10.1038/s41598-022-15369-2

**Published:** 2022-06-30

**Authors:** Phuoc Thien Phan, Mai Thanh Thai, Trung Thien Hoang, James Davies, Chi Cong Nguyen, Hoang-Phuong Phan, Nigel H. Lovell, Thanh Nho Do

**Affiliations:** 1 Graduate School of Biomedical Engineering, Faculty of Engineering, University of New South Wales (UNSW), Sydney, NSW 2052 Australia; 2 School of Mechanical and Manufacturing Engineering, Faculty of Engineering, University of New South Wales (UNSW), Sydney, NSW 2052 Australia

**Keywords:** Mechanical engineering, Actuators

## Abstract

The marriage of textiles with artificial muscles to create smart textiles is attracting great attention from the scientific community and industry. Smart textiles offer many benefits including adaptive comfort and high conformity to objects while providing active actuation for desired motion and force. This paper introduces a new class of programmable smart textiles created from different methods of knitting, weaving, and sticking fluid-driven artificial muscle fibers. Mathematical models are developed to describe the elongation-force relationship of the knitting and weaving textile sheets, followed by experiments to validate the model effectiveness. The new smart textiles are highly flexible, conformable, and mechanically programmable, enabling multimodal motions and shape-shifting abilities for use in broader applications. Different prototypes of the smart textiles are created with experimental validations including various shape-changing instances such as elongation (up to 65%), area expansion (108%), radial expansion (25%), and bending motion. The concept of reconfiguring passive conventional fabrics into active structures for bio-inspired shape-morphing structures is also explored. The proposed smart textiles are expected to contribute to the progression of smart wearable devices, haptic systems, bio-inspired soft robotics, and wearable electronics.

## Introduction

Rigid robots are effective when working in structured environments but encounter problems dealing with unknown contexts of changing environments, thus restricting their applications for searching or exploration. Nature always amazes us with numerous smart strategies to handle external factors and versatilities. For example, the tendrils of climbing plants perform multimodal motions such as bending and spiral twisting to explore the unknown environment to find suitable supports^1^. The Venus flytrap (*Dionaea muscipula*) is equipped with sensitive hairs on its leaves that upon trigger will snap shut to catch prey^2^. Shape-morphing or shape-shifting bodies from two-dimensional (2D) surfaces to three-dimensional (3D) shapes which mimic biological structures have become interesting research topics in recent years^3,4^. These soft robotic configurations alter their shapes to adapt to versatile environments, provide multimodal motions, and exert force to generate mechanical work. Their reaches have been expanded to a wide range of robotic applications, including deployable structures^5^, reconfigurable and self-folding robots^6,7^, biomedical devices^8^, locomotion^9,10^, and stretchable electronics^11^.

Many studies have been conducted to develop a programmable planar sheet that transforms into a complex 3D structure upon activation^3^. A simple idea to generate shape-shifting structures is to combine layers of different materials that yield bending and wrinkle motions when triggered by stimuli^12,13^. This concept has been implemented by Janbaz et al.^14^ and Lee et al.^15^ to produce thermally-responsive multimodal shape-shifting robots. Origami-based structures incorporated with stimuli-responsive elements have been exploited to create complex 3D structures^16–18^. Inspired by the morphogenesis of biological structures, Emmanuel et al. created shape-morphing elastomers by arranging airways inside a rubber surface, which transform into complex arbitrary 3D shapes upon pressurization^19^.

Integrating textiles or fabrics into shape-morphing soft robots is another emerging conceptual design that attracts intensive interest. Textiles are soft and flexible materials made from yarns by interlacing techniques such as knitting, weaving, braiding, or knotting. Textiles have amazing characteristics, including flexibility, conformability, stretchability, and breathability, making them extremely popular in every aspect of life from clothing to medical applications^20^. There are three broad approaches to incorporating textiles into robotics^21^. The very first approach is to use textiles as passive substrates or the base to house other components. In this circumstance, passive textiles provide a comfortable fit to the users while carrying rigid components (motors, sensors, power suppliers). Most soft wearable robots or soft exoskeletons belong to this approach. For example, soft wearable exoskeletons for walking assistance^22^ and elbow joint assistance^23–25^, a soft wearable glove for hand and finger assistance^26^, and bio-inspired soft robots^27^.

The second approach is to use textiles as passive and constrained components of soft robotic devices. Textile-based actuators fall into this category where textiles are normally constructed as external containers to constrain inner soft tubes or bladders which form soft fibre-reinforced actuators. Upon pressurization by an external pneumatic or hydraulic source, these soft actuators exert shape-change, either elongating, bending, or twisting, according to their initial compositions and configurations. For example, Thalman et al. introduced an ankle–foot orthosis exosuit made from a series of fabric pouches to assist in plantar flexion for gait rehabilitation^28^. Textile layers with different stretchability could be combined to produce anisotropic motions^29^. OmniSkins—soft robotic skins made of various soft actuators and substrate materials could allow the transformation of passive objects into multifunctional active robots that could perform multimodal locomotion and deformations for various applications^30^. Zhu et al. developed fluidic fabric muscle sheets that produced elongating, bending, and various shape-shifting motions^31^. Buckner et al. integrated functional fibers into conventional fabrics to create robotic fabrics that had multiple functions such as actuation, sensing, and variable stiffness^32^. Other approaches in this category can be found in these works^21,33–35^.

The latest approach which leverages excellent textile properties in the soft robotic field is to use active yarns or stimuli-responsive filaments to construct smart textiles by using traditional textile-making approaches such as braiding, knitting, and weaving techniques^21,36,37^. Depending on material compositions, the active yarns induce shape-change when triggered by electrical, thermal, or pressure inputs, resulting in textile deformation. In this approach, the shape-change of textiles happens at the interior level (yarns) rather than the exterior level when integrating conventional textiles into soft robotic systems. Therefore, smart textiles offer great controllability in terms of multimodal motion, programmable shape-shifting, stretchability, and stiffness tuneability. For example, shape memory alloys (SMAs) and shape memory polymers (SMPs) could be incorporated into fabrics to actively control their shapes by thermal excitation such as a roll-up hemline^38^, wrinkle recovery^36,39^, tactile and haptic feedback^40,41^, and self-fitting wearables^42^. However, the use of thermal heating and cooling resulted in slow response, complex cooling, and control. Recently, Hiramitsu et al. implemented thin McKibben muscles^43,44^ (a type of pneumatic artificial muscles) as warps to create multiple forms of active textiles by changing woven structures^45^. Although this approach offers high force, it was limited at the expansion rate (< 50%) and could not achieve a small size (< 0.9 mm in diameter) due to the nature of the McKibben muscle. In addition, it found challenges to form smart textile patterns from the knitting method, which requires a sharp bending angle. To form a larger array of smart textiles, Maziz et al. developed electroactive textiles for wearable devices by knitting and weaving electrically responsive polymer yarns^46^.

Recent years have witnessed the emergence of a new class of thermally-responsive artificial muscles constructed from highly twisted inexpensive polymer fibers^47,48^. These fibers are commercially available and easy to incorporate into machine-knitted or -woven to produce affordable smart garments. Despite advances, these new thermal smart textiles are limited with slow response time due to the requirement of heating and cooling (e.g. thermally controlled textiles) or difficulties to fabricate complex knitting and weaving patterns that can be programmed to form desired deformations and motions such as radial expansion, shape transformation from 2 to 3D or bidirectional expansion as we propose here.

To overcome these aforementioned challenges, this paper introduces a new class of fluid-driven smart textiles formed from our recently soft artificial muscle fibers (AMFs)^49–51^. The AMFs are highly flexible, scalable with sizes that can be scaled down to 0.8 mm in diameter, long length (at least 5000 mm) which offers a high-aspect ratio (length-per-diameter) as well as high elongation (at least 245%), high energy efficiency, and fast response at least 20 Hz). To create smart textiles, we employ AMFs as active yarns to form 2D active muscle sheets by knitting and weaving techniques. We quantitatively examine the expansion ratio and contraction force of these smart textiles with respect to applied input fluid volume and pressure. Analytical models have been developed to establish the elongation-force relationship of knitted and woven sheets. We also introduce several techniques to mechanically program the smart textiles to achieve multimodal motion including bidirectional elongation, bending, radial expansion, and growing ability from 2 to 3D. To demonstrate the capability of our approaches, we also integrate the AMFs into commercial fabrics or textiles to reconfigure them from passive to active structures that can induce different deformations. We also demonstrate this concept via several experimental testbeds including programmable bending filaments to achieve desired letters and bio-inspired shape-morphing structures in the form of objects such as a butterfly, four-legged structures, and a flower with complex motions.

## Results

### Smart textile composition and configurations

A textile is a flexible two-dimensional structure created by interlocking one-dimensional filaments such as yarns, threads, and fibers. Textiles are one of humankind’s oldest technologies, being widely used in every aspect of life thanks to their comfort, adaptability, breathability, appearance, and protection. Smart textiles (also known as smart garments or robotic fabrics) have increasingly been used in research studies because of their vast potential in robotic applications^20,52^. Smart textiles are expected to enhance human experiences to interact with soft bodies, opening a paradigm shift in the field where a thin and flexible piece of fabric can be controlled in motion and force to perform specific tasks. In this work, we explore two methods to produce smart textiles based on our recent AMFs:^49^ (1) using the AMFs as active yarns to construct smart textiles by traditional textile-making techniques; (2) directly sticking the AMFs into conventional fabrics to induce desired motion and deformation.

**Figure 1:**
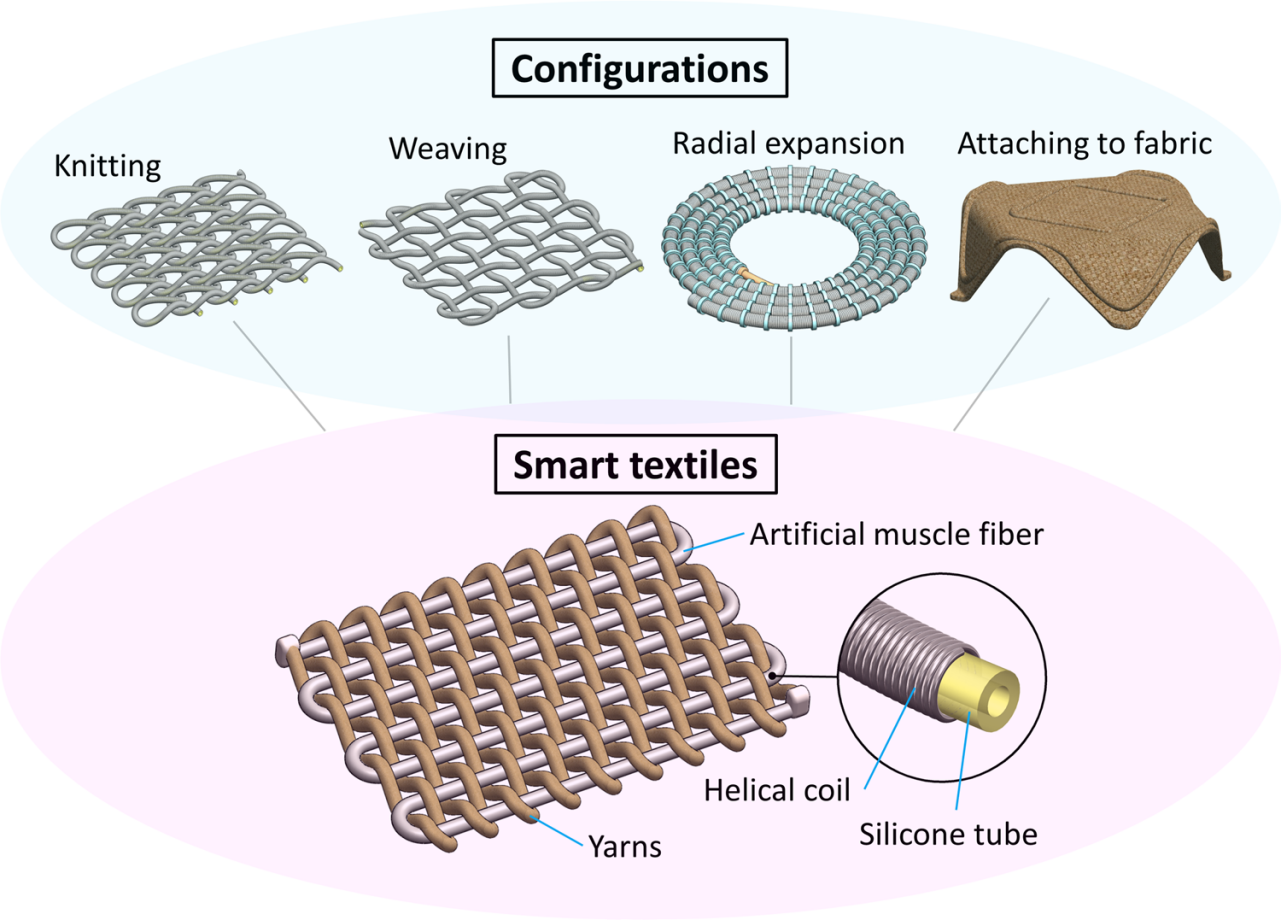
Different approaches to create smart textiles from artificial muscle fibers.

The AMFs consist of an inner silicone tube to receive hydraulic power and an outer helical coil to constrain its radial expansion. Therefore, AMFs produce longitudinal elongation upon pressurization and subsequently exert contraction force when releasing the pressure to return to the initial length. They possess similar characteristics as traditional fibers including flexibility, small diameter, and long length. However, the AMFs surpass their traditional counterparts by being active and controllable in terms of motion and force. Motivated by recent fast-paced developments in the smart textile field, we introduce here four main approaches to producing smart textiles by implementing the AMFs into long-established fabric-making techniques (Fig. 1).

The first approach is knitting. We utilize the weft knitting technique to produce an active knitting sheet that can expand in one direction upon hydraulic pressurization. Knitting sheets are highly elastic and highly stretchable but more prone to unravelling than weaving sheets. One AMF can form a single course or an entire knitting sheet depending on control approaches. Besides the planar sheet, tubular knitting patterns are applicable for AMF to make hollow structures. The second approach is weaving where we make use of two AMFs as warp and weft to form a rectangular weaving sheet that can exert independently bidirectional expansion. Weaving sheets offer a greater control degree (two directions) compared to knitting sheets. We also weave a single AMF with conventional yarns to produce a simpler weaving sheet that can only expand in one direction. The third approach is radial expansion—a variant of the weaving technique where instead of being rectangularly arranged, the AMF is aligned in a spiral shape with yarns providing radial constraint. In this case, the weaving sheet will expand radially when receiving input pressure. The fourth approach is to stick the AMFs to a passive fabric sheet to create bending motions in desired directions. We reconfigure a passive fabric sheet into an active one by routing an AMF around its boundary. This programmable characteristic of the AMF opens numerous possibilities for bio-inspired shape-morphing soft structures where we can turn passive objects into active ones. This approach is simple, easy, and fast but may compromise the prototypes’ durability. Readers can refer to other approaches from the literature which detailed the pros and cons of the performance of each type of textile^21,33–35^.

### AMF configurations to produce smart textiles

Most filaments or yarns which produce conventional textiles comprise a passive structure. In this work, we take advantage of our previously developed AMFs that can be made meters in length with sub-millimeter diameters, replacing the conventional filaments of the passive textiles by the AFMs to form smart and active textiles for broader applications. The following sections describe detailed fabrication methods to produce smart textile prototypes as well as introduce their basic characteristics and behaviors.

#### Knitting sheet

**Figure 2:**
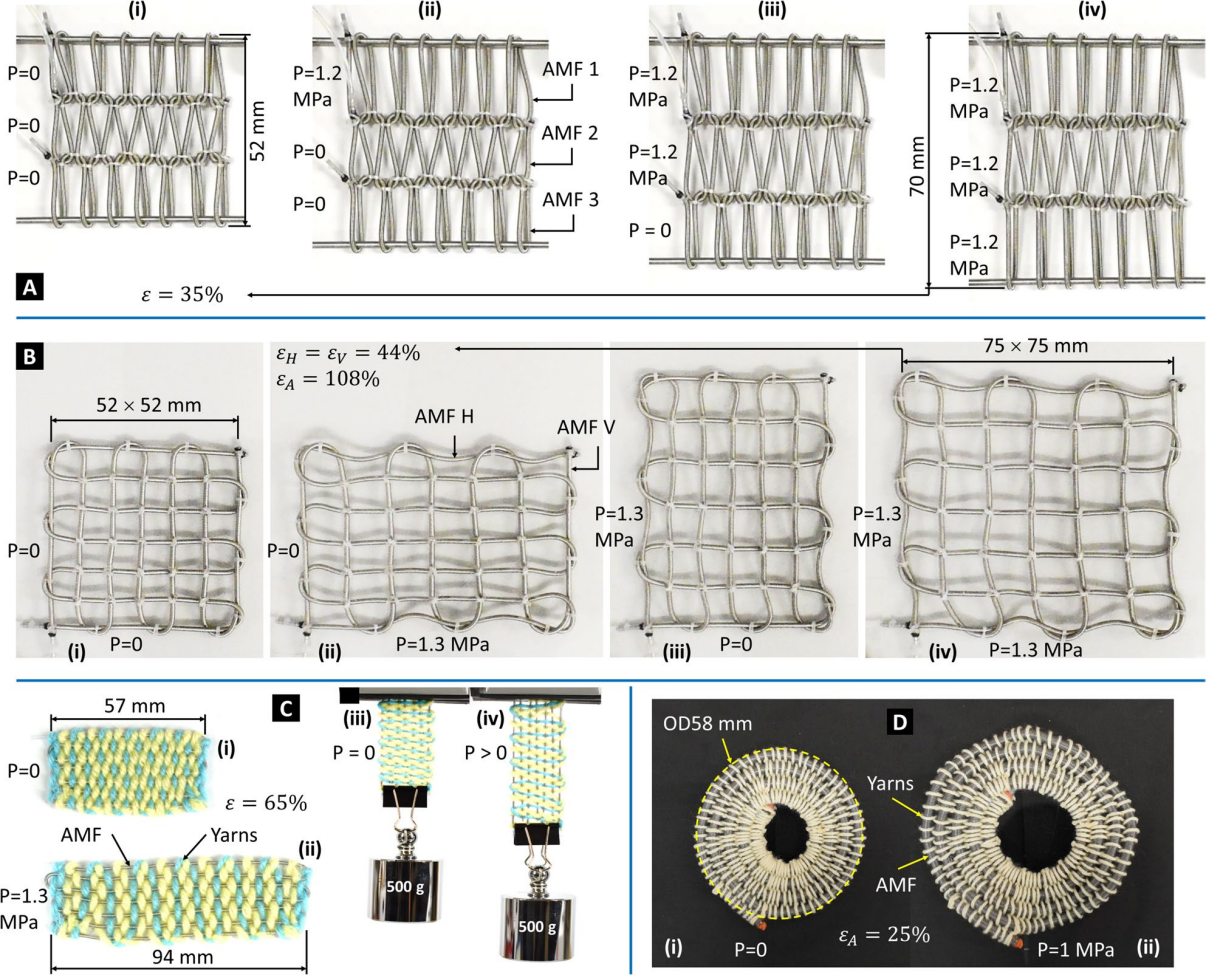
Different prototypes of smart textiles are achieved by varying AMF configurations. (**A**) Knitting sheet made from three AMFs. (**B**) Bidirectional weaving sheet made from two AMFs. (**C**) A unidirectional weaving sheet made from one AMF and acrylic yarns could lift a 500 g load, which is 192 times heavier than its mass (2.6 g). (**D**) Radial expansion structure made from a single AMF and cotton yarns as radial constraints. Detailed specifications can be found in the “Methods” section.

We manually fabricated a knitting sheet from three AMFs using the weft knitting technique (Fig. 2A). Material selections and detailed specifications of AMFs and prototypes can be found in the “Methods” section. Each AMF followed a meandering path (also known as a course) that formed symmetric loops. The loops of each course were secured with the loops of the courses just above and below them. Loops of the same column that were perpendicular to the course were grouped into a wale. Our knitting prototype consisted of three courses with seven loops for each course (or seven wales). The loops of the top and bottom courses were not secured so that we could connect them to corresponding metal rods. The knitting prototype was more prone to unravel than conventional knitting fabric because of the higher stiffness of AMFs compared to conventional yarns. Therefore, we constrained loops of adjacent courses by a thin elastic string.

Although the meandering loops of the knitting sheet could be stretched in different directions, our knitting prototype primarily expanded in the wale direction upon pressurization owing to the constraint in the course direction. The elongation of each AMF contributed to the overall area expansion of the knitting sheet. Depending on specific requirements, we could control three AMFs independently from three different fluid sources (Fig. 2A) or simultaneously from a single fluid source via a 1-to-3 fluid distributor. Figure 2A shows one instance of the knitting prototype that expanded 35% of its initial area when pressurizing (1.2 MPa) three AMFs simultaneously. It is noted that the AMF achieved a high elongation of at least 250% of its initial length^49^ and therefore the expansion ratio of the knitting sheet can be made higher than that of the current version.

#### Weaving sheet

We also created a bidirectional weaving sheet formed from two AMFs by using the plain weaving technique (Fig. 2B). The warp and weft AMFs were interlaced at right angles to form a simple criss-cross pattern. Our weaving prototype was classified as balanced plain weaves because both warp and weft were made of filaments of the same size (detailed specifications are shown in the “Methods” section). Unlike conventional yarns which are capable of sharp folds, the used AMFs require a certain bending radius when turning back for another line of the weaving pattern. As a result, the weaving sheet made from the AMFs is less dense compared to conventional weaving textiles. The minimum bending radius of AMFs type S (OD 1.49 mm) is 1.5 mm. As an illustration, our weaving prototype presented in this paper had a pattern of 7 × 7 lines where each cross was stabilized by a knot made of a thin elastic string. A higher number of lines can be achieved using the same weaving technique.

The weaving sheet expanded its area toward the warp or weft direction when the corresponding AMF received fluid pressure. Therefore, we controlled the weaving sheet dimension (length and width) by independently varying the amplitude of applied input pressure to the two AMFs. Figure 2B shows the weaving prototype that expanded 44% of its initial area when pressurizing (1.3 MPa) one AMF at a time. An area expansion of 108% was achieved when simultaneously pressurizing the two AMFs.

We also fabricated a unidirectional weaving sheet formed from a single AMF as warp and acrylic yarns as weft (Fig. 2C). The AMF was arranged in a seven-line zigzag whereas the yarns interlaced these AMF lines to form a rectangular textile sheet. This weaving prototype was denser than the one in Fig. 2B thanks to the soft acrylic yarns that easily filled the entire sheet. Since we used only one AMF as the warp, the weaving sheet can only expand toward the warp direction upon pressurization. Figure 2C shows one instance of the weaving prototype that expanded 65% of its initial area when pressurizing (1.3 MPa). In addition, this weaving sheet (which weighed 2.6 g) could lift a load of 500 g which is 192 times heavier than its mass.

#### Radial expansion with circular weaving sheet

Instead of arranging an AMF in a zigzag layout to create a rectangular weaving sheet, we made a planar spiral shape of an AMF and then constrained it radially with cotton yarns to create a circular weaving sheet (Fig. 2D). The high stiffness of the AMF restrained it from filling the centermost area of the circular sheet. However, this filling can be done with elastic yarns or stretchable fabrics. When receiving hydraulic pressure, the AMF transformed its longitudinal elongation into the radial expansion of the sheet. It is also worth noting that both the outer and inner diameters of the spiral shape are expanded, due to the radial constraint of the yarns. Figure 2D shows the shape of the circular sheet that expanded 25% of its initial area under applied hydraulic pressure of 1 MPa.

#### Fabric reconfiguration

**Figure 3:**
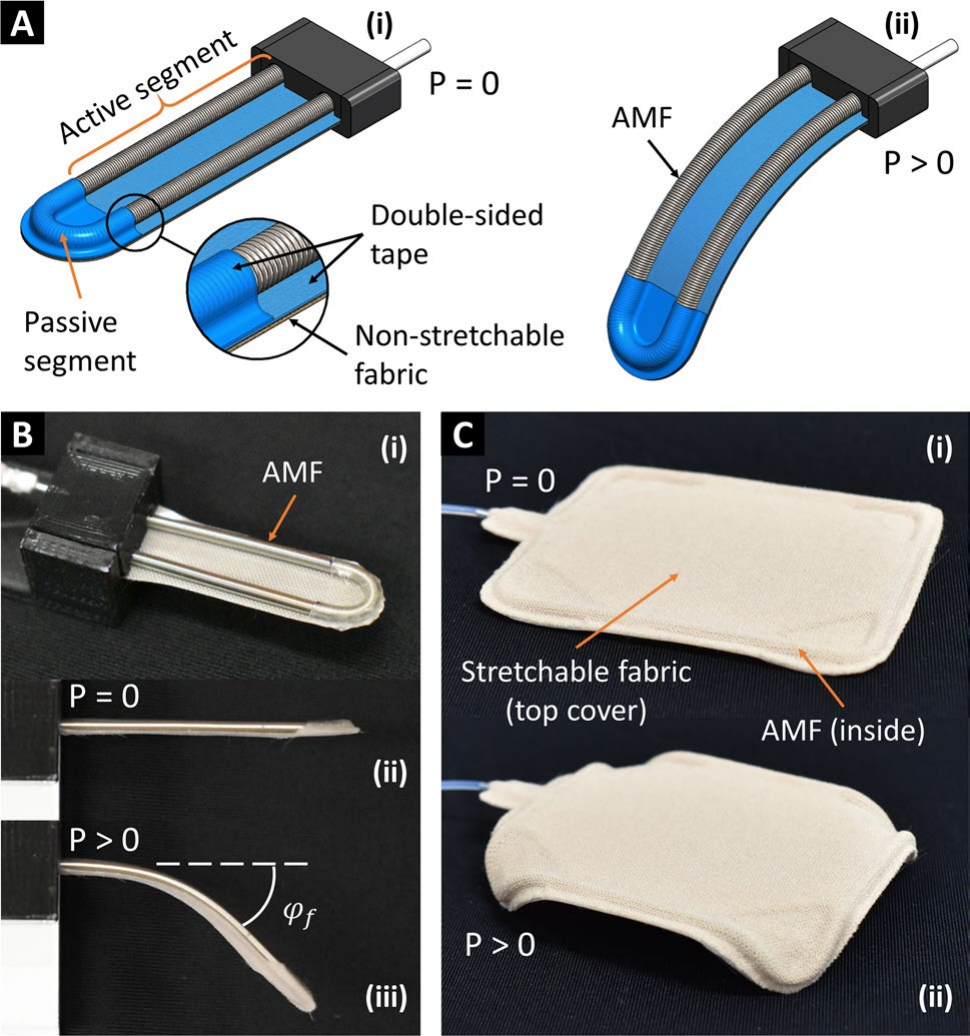
Fabric reconfiguration by sticking AMFs to conventional fabrics. (**A**) Design concept of bending actuators made by sticking a folding AMF to a non-stretchable fabric. (**B**) Bending actuator prototype. (**C**) Reconfiguring a rectangular fabric into an active four-legged robot. Non-stretchable fabric: plain-woven cotton muslin; stretchable fabric: polyester. Detailed specifications can be found in the “Methods” section.

We introduce here the second method to create smart textiles where we stick an AMF to a piece of planar fabric to reconfigure it from a passive to an active, controllable structure. The design concept of a bending actuator is illustrated in Fig. 3A where an AMF is folded at its midpoint and stuck to a strip of non-stretchable fabric (cotton muslin fabric) using double-sided tape as a bonding element. Upon pressurization, the top part of the AMF elongates freely whereas the bottom part is constrained by the tape and fabric, resulting in a bending motion of the strip toward the fabric side. We can deactivate any segment at any location of the bending actuator by simply putting a piece of tape on top of it. The deactivated segments are unable to exert any motion and become passive segments.

We fabricated several bending actuator prototypes with different lengths and hydraulically pressurized them to generate the bending motion (Fig. 3B). It is noted that the AMF can be arranged in a straight line or folded to form multiple lines before sticking to the fabric to create a bending actuator with the corresponding number of lines. We also reconfigured a passive fabric sheet into an active four-legged structure (Fig. 3C) where we routed the boundaries of rectangular non-stretchable fabric (cotton muslin fabric) with an AMF. The AMF was stuck to the fabric with the help of a piece of double-sided tape. The middle segments of each edge were taped to become passive, leaving four corners active. A top cover made of stretchable fabric (polyester) was optional. The four corners of the fabric were bent down (looked like legs) when pressurized.

### Characteristics of smart textiles

#### Input pressure versus output elongation and force

**Figure 4:**
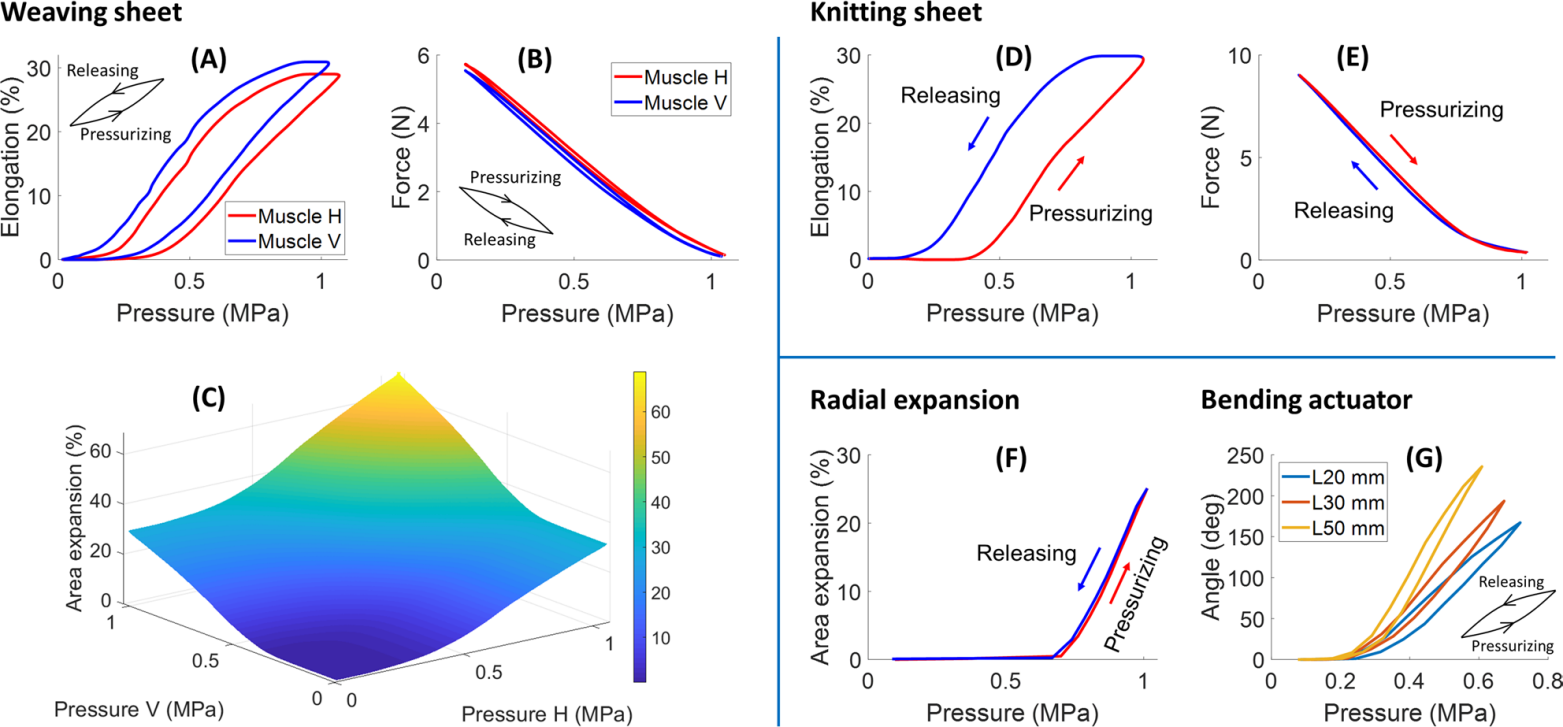
Characteristics of smart textile configurations. (**A**,**B**) Hysteresis profiles of input pressure and output elongation and force of the weaving sheet. (**C**) Area expansion of the weaving sheet. (**D**,**E**) Relationship between input pressure and output elongation and force of the knitting sheet. (**F**) Area expansion of the radial expansion structure. (**G**) Bending angles of three different lengths of bending actuators.

We built a testing platform to quantitatively examine the characteristics of the developed smart textiles (see the “Methods” section and Supplementary Fig. S1). Since all specimens were made of AMFs, the overall trend of experimental results (Fig. 4) agrees with the AMF’s fundamental characteristic where the input pressure has a proportional relationship with output elongation and a reverse proportional relationship with contraction force. However, these smart textiles have their own distinctive features that represent their particular configurations.

Each AMF of the weaving sheet received 1 MPa input pressure to generate approximately 30% elongation (Fig. 4A). We selected this threshold for the whole experiment for several reasons: (1) to create a substantial elongation (around 30%) to highlight their hysteresis profile, (2) to prevent unexpected damage or malfunction resulting from cyclic motion and reusable prototypes for different experiments under high fluid pressure. A dead zone was clearly visible, where the weaving sheet remained static until input pressure reached 0.3 MPa. The pressure-elongation hysteresis chart showed a wide gap between the pressurizing and releasing phases, indicating a significant energy loss when the weaving sheet changes its motion from expansion to contraction. (Fig. 4A). The weaving sheet could exert a contraction force of 5.6 N after receiving 1 MPa input pressure (Fig. 4B). The pressure-force hysteresis chart also showed that the releasing curve almost overlapped the pressurizing curve. The weaving sheet’s area expansion relies on the pressure amplitudes supplied to each of the two AMFs, which are shown in the three-dimensional surface plot (Fig. 4C). Experiments also revealed that the weaving sheet could generate an area expansion of 66% when its warp and weft AMFs simultaneously received 1 MPa hydraulic pressure.

Experimental results of the knitting sheet have a similar pattern to that of the weaving sheet, including the wide hysteresis gap in the pressure-elongation chart and the overlapping curves in the pressure-force relation. The knitting sheet generated 30% elongation and subsequently exerted a contraction force of 9 N when receiving 1 MPa input pressure (Fig. 4D,E).

In the case of the circular weaving sheet, there was an expansion from its initial area to 25% after receiving 1 MPa fluid pressure (Fig. 4F). There was a substantial dead zone of input pressure until 0.7 MPa before the specimen started to expand. This large dead zone is expected because the specimen was made of a larger AMF that required a higher pressure to overcome its initial tension. Figure 4F also showed that the releasing curves almost overlapped the pressurizing curves, denoting inconsiderable energy loss when switching the circular sheet motions.

Experimental results for three bending actuators (fabric reconfiguration) showed that their hysteresis profiles share similar patterns (Fig. 4G), in which they underwent a dead zone of input pressure until 0.2 MPa before rising. We supplied the same volume of fluid (0.035 mL) to the three bending actuators (L20, L30, and L50 mm). However, each actuator experienced different pressure peaks and generated different bending angles. The L20 and L30 mm actuators experienced 0.72 and 0.67 MPa input pressure to reach a bending angle of 167° and 194°, respectively. The longest bending actuator (L50 mm) experienced 0.61 MPa pressure to achieve the largest bending angle of 236°. The pressure-angle hysteresis chart also showed a relatively wide gap between the pressurizing and releasing curves of all three bending actuators.

The relationship between input volume and output characteristics (elongation, force, area expansion, bending angle) of the above smart textile configurations can be found in Supplementary Fig. S2.

#### Elongation-force relationship of knitting and weaving sheets

Experimental results in the previous section clearly demonstrated the proportional relation between the applied input pressure and the output elongation of specimens made of AMFs. The more pressure the AMF receives, the more elongation it generates and the more elastic energy it accumulates. Consequently, the more contraction force it exerts. The results also revealed that the specimens reached their maximum contraction force when input pressure was completely withdrawn. This section aims to establish the direct relationship between elongation and maximum contraction force of knitting and weaving sheets by both analytical models and experimental validation.

The maximum contraction force *F*_*out*_ of a single AMF (when input pressure *P* = 0) has been presented in reference^49^, which is reintroduced as follows:

1 $${F}_{out}=\alpha E{A}_{0}\left(1-\frac{1}{1+x/{l}_{i}}\right)+kx,$$

where *α*, *E*, *A*_0_ represents the stretch ratio, Young’s modulus, and cross-sectional area of the silicone tube, respectively; *k* is the stiffness coefficient of the helical coil; *x* and *l*_*i*_ are the displacement and initial length of the AMF, respectively.

**Figure 5:**
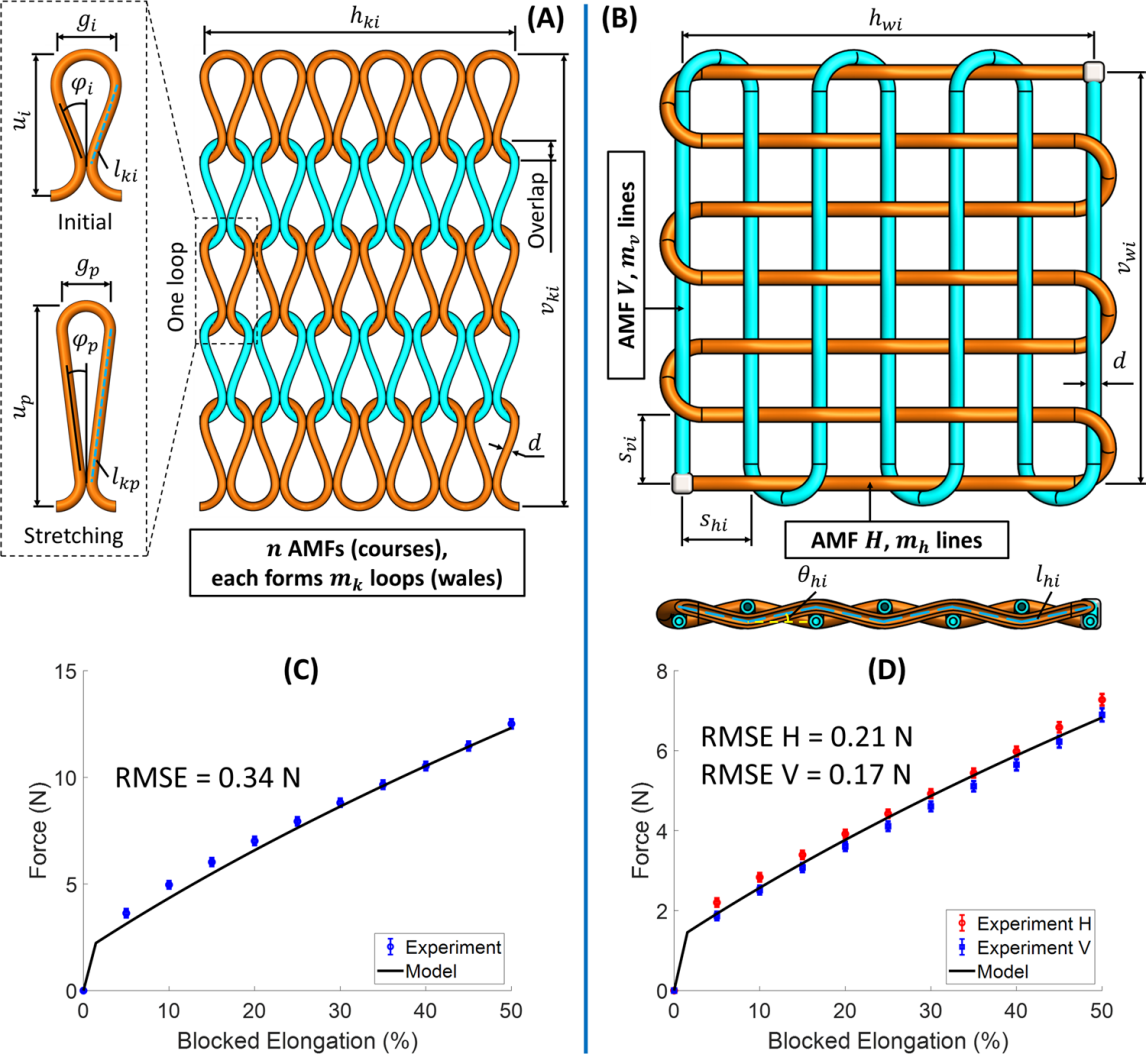
Analytical models to establish the elongation-force relationship. (**A**,**B**) Analytical model illustration for knitting and weaving sheets, respectively. (**C**,**D**) Comparison between analytical models and experimental data for knitting and weaving sheets, respectively. *RMSE* root mean square error.

Adapting Eq. (1) to the case of knitting and weaving sheets (Fig. 5A,B). The contraction force of a knitting sheet *F*_*kv*_ and a weaving sheet *F*_*wh*_ are expressed by Eqs. (2) and (3), respectively.

2 $$ \begin{aligned} & F_{kv} = 2m_{k} \left[ {\alpha EA_{0} \left( {1 - \frac{1}{{1 + x_{kv} /l_{ki} }}} \right) + \left\{ {\begin{array}{*{20}l} {qx_{kv} , \qquad\quad  \varepsilon_{kv} \le 2\% } \\ {kx_{kv} + F_{0} ,\quad  \varepsilon_{kv} > 2\% } \\ \end{array} } \right.} \right]\cos \varphi_{p} \\ & {\text{Continuity}}\,{\text{condition:}}q = k + F_{0} /x_{kv} , \quad\varepsilon_{kv} = 2\% , \\ \end{aligned} $$

3 $$ \begin{aligned} & F_{wh} = m_{h} \left[ {\alpha EA_{0} \left( {1 - \frac{1}{{1 + x_{wh} /l_{hi} }}} \right) + \left\{ {\begin{array}{*{20}l} {qx_{wh} , \qquad \quad \varepsilon_{wh} \le 2\% } \\ {kx_{wh} + F_{0} ,\quad \varepsilon_{wh} > 2\% } \\ \end{array} } \right.} \right]\cos \theta_{hp} \\ & {\text{Continuity}}\,{\text{condition:}}q = k + F_{0} /x_{wh} ,\quad \varepsilon_{wh} = 2\% , \\ \end{aligned} $$

where *m*_*k*_ is the number of wales and *φ*_*p*_ is the looping angle at the pressurizing phase of the knitting sheet (Fig. 5A); *m*_*h*_ is the number of lines and *θ*_*hp*_ is the interlocking angle at the pressurizing phase of the weaving sheet (Fig. 5B); *ε*_*kv*_ and *ε*_*wh*_ are the strain of the knitting sheet and the weaving sheet, respectively; *F*_*0*_ is the initial tension of the helical coil. Detailed derivations of Eqs. (2) and (3) can be found in the Supporting Information.

To validate the developed models, we conducted elongation-force experiments using the knitting specimen in Fig. 2A and the weaving specimen in Fig. 2B. The contraction force was collected at each blocked elongation from 0 to 50% with a 5% increment. Mean values and standard deviation of five trials were plotted in Fig. 5C (for knitting) and Fig. 5D (for weaving). The analytical model curve was governed by Eqs. (2) and (3) with parameters as shown in Table 1. The results show that the analytical models closely followed experimental data for the entire elongation range with a root mean square error (RMSE) of 0.34 N for knitting, 0.21 N for weaving AMF H (horizontal direction), and 0.17 N for weaving AMF V (vertical direction).

**Table 1:** Parameters of analytical models.

	Helical coil		Silicone tube	
AMF	*d*	1.49 (mm	*α*	0.9
	*k*	0.011 (N/mm)	*E*	1.648 (MPa)
	*F*_*0*_	0.126 (N)	*A*_*0*_	0.8026 (mm^2^)

	Knitting		Weaving	
Configuration	*n*	3	*m*_*h*_	7
	*m*_*k*_	7	*m*_*v*_	7
	*h*_*ki*_	54 (mm)	*h*_*wi*_	52 (mm)
	*v*_*ki*_	52 (mm)	*v*_*wi*_	52 (mm)
	*ε*_*kv*_	0–50 (%)	*ε*_*wh*_	0–50 (%)

*k* and *F*_*0*_ at length 50 mm, *E* at 100% strain.

### Shape-programmability of smart textiles

In addition to the basic motions, the proposed smart textiles can be mechanically programmed to provide more complex motions such as s-shape bending, radial compression, and shape-shifting from 2 to 3D. We introduce here several techniques to program a planar smart textile into desired structures.

#### Unidirectional weaving sheet

**Figure 6:**
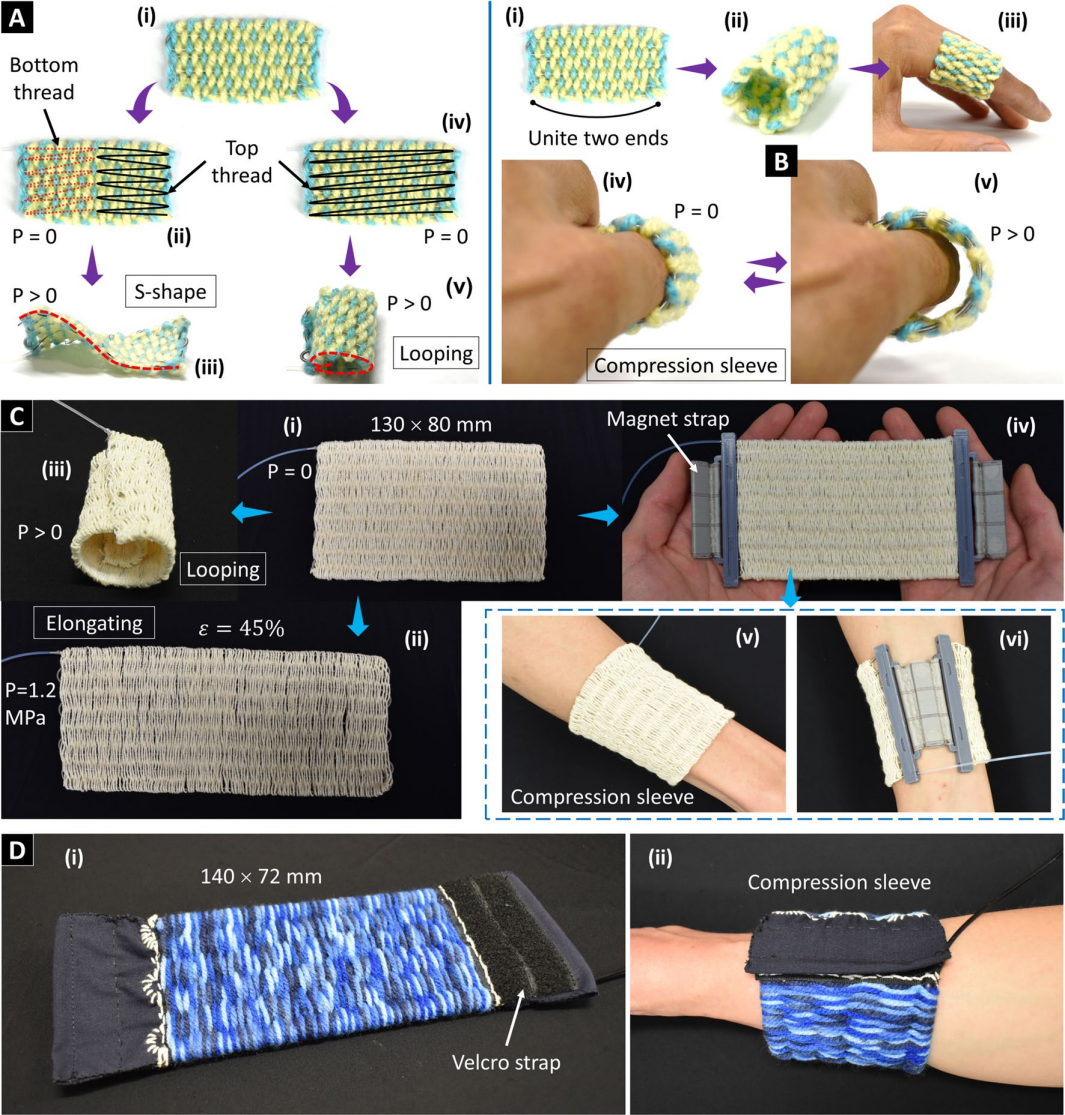
Capabilities of unidirectional weaving sheets. (**A**) Shape programmability by sewing thread to produce shape-shifting structures. (**B**) Compression sleeve for a finger. (**C**) Another weaving sheet embodiment and its implementation as a forearm compression sleeve. (**D**) Another compression sleeve prototype made of an AMF type M, acrylic yarns, and a Velcro strap. Detailed specifications can be found in the “Methods” section.

Alongside area expansion in a straight direction, unidirectional weaving sheets can be mechanically programmed to produce multimodal motion (Fig. 6A). We reconfigured the weaving sheet elongation into bending motion by constraining its one face (either top or bottom) using sewing thread. The sheet tends to bend toward the constraining face upon pressurization. Figure 6A shows two instances of the weaving sheet, where it transformed into an s-shape when constraining one half at the top face and the other half at the bottom face. Alternatively, when constraining only one entire face a loop bending motion could be generated. The unidirectional weaving sheet can also be implemented as a compression sleeve by uniting its two ends to form a tubular structure (Fig. 6B). The sleeve could enclose the human index finger and provide compression force, which is massage therapy to relieve pain or improve blood circulation. It can be scaled up to fit other body parts such as arms, thighs, and legs.

Figure 6C presents another embodiment of the unidirectional weaving sheets made of a single AMF and cotton yarns. The sheet could generate 45% area expansion (at 1.2 MPa) or induce a looping motion upon pressurization. We also implemented the sheet to create a compression sleeve for the forearm by attaching a magnetic strap to the sheet’s ends. Another compression sleeve prototype for the forearm is shown in Fig. 6D where the unidirectional weaving sheet was made of an AMF type M (see the “Methods” section) and acrylic yarns to generate a stronger compression force. We equipped the sheet’s ends with Velcro straps to facilitate attachment and adapt to various arm sizes.

#### Bidirectional weaving sheet

**Figure 7:**
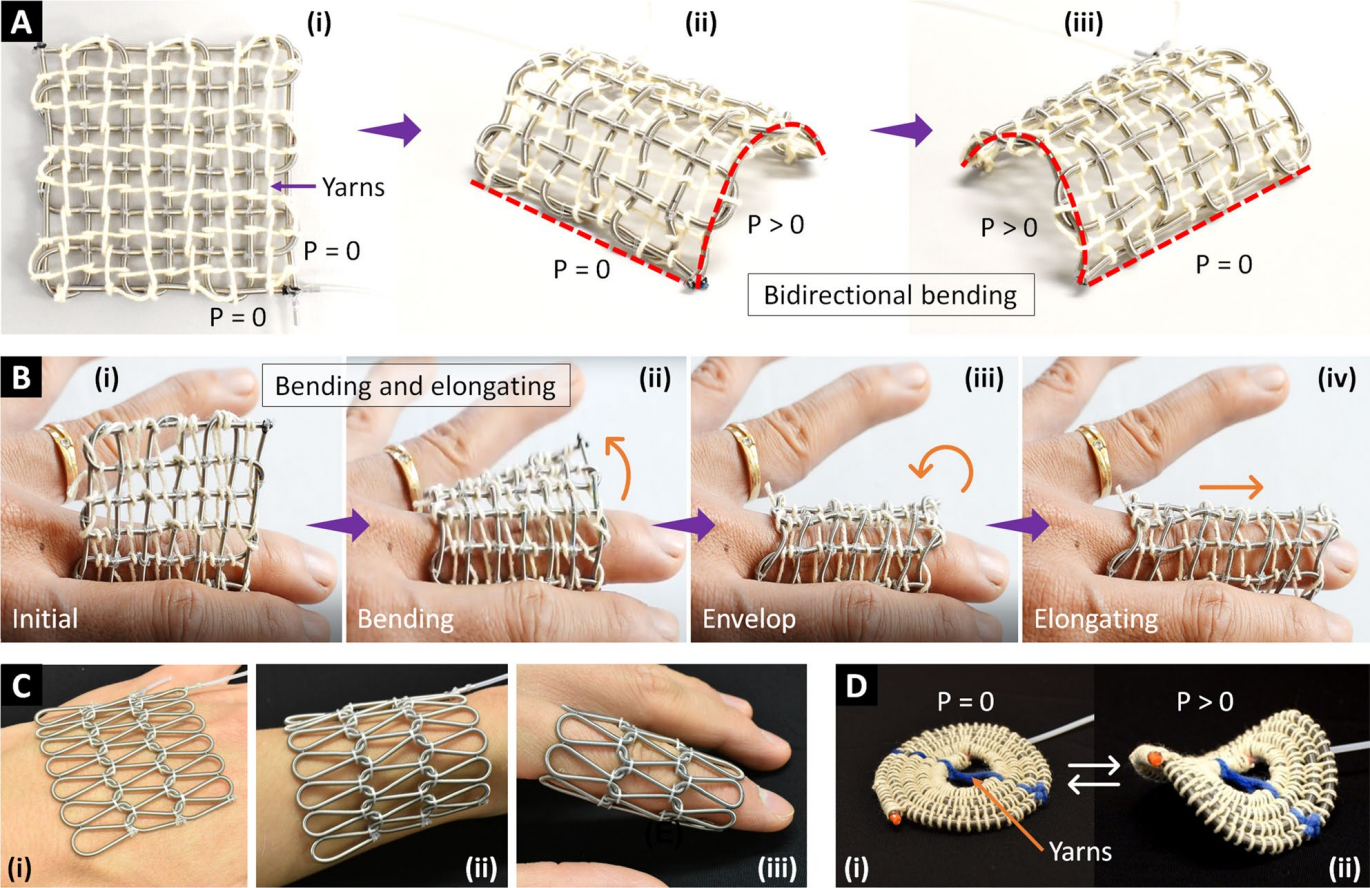
Capabilities of a bidirectional weaving sheet, knitting sheet, and radial expansion structure. (**A**) Bidirectionally constraining a bidirectional weaving sheet to produce bidirectional bending. (**B**) Unidirectionally constraining a bidirectional weaving sheet to produce bending and elongating. (**C**) Highly conformable knitting sheet that could adapt to various surface curvatures or even form a tubular structure. (**D**) Constraining the centerline of a radial expansion structure to form a hyperbolic paraboloid shape (a potato chip).

The constraining technique to transform straight elongation into bending motion is also applicable for the bidirectional weaving sheet. We interlaced cotton yarns to one face of the weaving sheet in both warp and weft directions to impede its expansion (Fig. 7A). Therefore, when two AMFs independently received hydraulic pressure, the sheet exerted a bidirectional bending motion, forming arbitrary 3D structures. On the other approach, we used inextensible yarns to constrain one direction of the bidirectional weaving sheet (Fig. 7B). As a result, the sheet could generate independent bending and elongating motion when pressurizing the corresponding AMFs. Figure 7B showed one instance in which the bidirectional weaving sheet was controlled to envelop two-thirds of the human finger by bending motion and then extend its length to cover the remaining by elongating motion. The bidirectional motion of the sheet may benefit fashion design or smart garment development.

#### Knitting sheet

We joined two adjacent loops of the top and bottom courses of the knitting sheet by sewing thread to secure it from unraveling (Fig. 7C). Consequently, the knitting sheet is entirely flexible and highly conformable to various surface curvatures such as skin surfaces of the human hand and arm. We also created a tubular structure (sleeve) by uniting two ends of the knitting sheet in the course direction. The sleeve nicely embraced the human index finger (Fig. 7C). The meandering features of the knitting sheet offer great conformability and deformability, facilitating its use in smart garments (gloves, compression sleeves) that provide the wearers with comfort (by adaptability) and therapeutic effect (by compression force).

#### Radial expansion

Besides 2D radial expansion in multiple directions, the circular weaving sheet can also be programmed to form a 3D structure. We constrained the centerline of the circular weaving sheet with acrylic yarns to disrupt its uniform radial expansion. As a result, the initial planar shape of the circular weaving sheet was transformed into a hyperbolic paraboloid-like shape (or a potato chip) upon pressurization (Fig. 7D). This shape-shifting capability can be implemented as a lifting mechanism, optical lens, legs for locomotion robots, or maybe useful for fashion design and bio-mimicking robots.

#### Fabric reconfiguration

We have developed a simple technique to create a bending actuator—by sticking an AMF to a strip of non-stretchable fabric (Fig. 3). We leveraged this concept to create shape-programmable filaments, in which we can strategically allocate multiple active and passive segments in a single AMF to produce the desired shape. We fabricated and programmed four active filaments that could transform their shapes from straight lines into letters (UNSW) upon pressurization (Supplementary Fig. S4). This simple technique enables the shape-shifting capability of AMFs to turn 1D lines into 2D shapes and possibly 3D structures.

**Figure 8:**
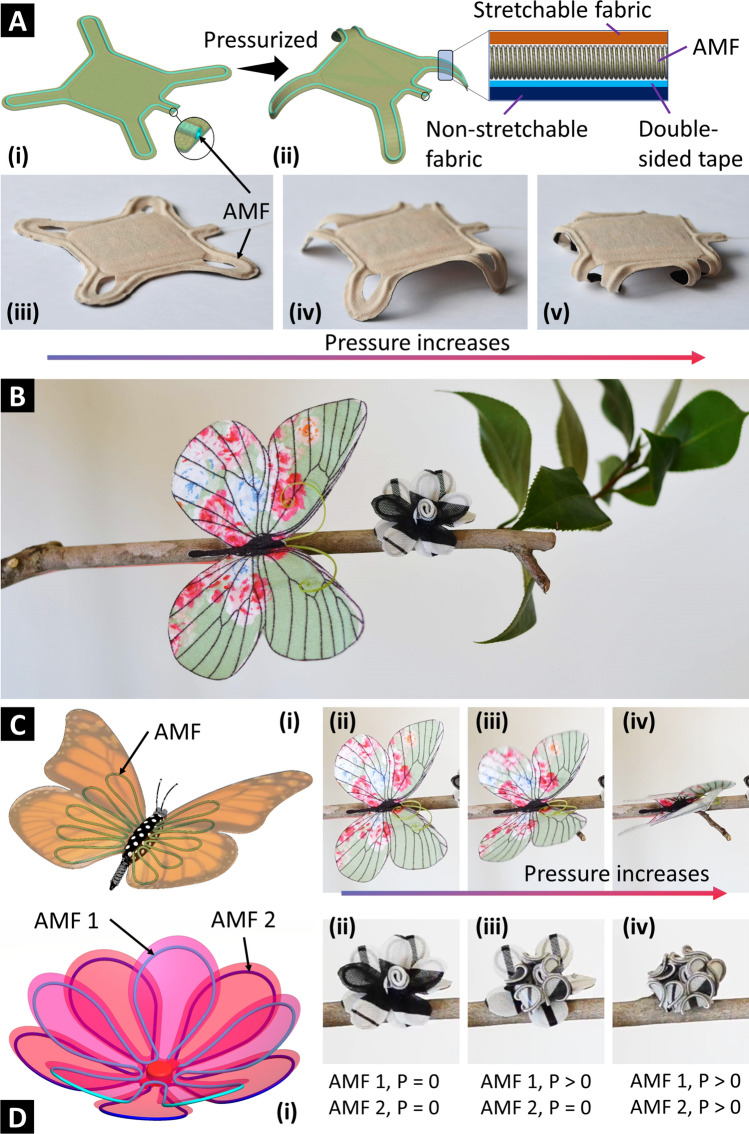
Fabric reconfiguration to produce shape-morphing structures. (**A**) Sticking an AMF to passive fabric sheet’s boundaries to turn it into a controllable four-legged structure. (**B**–**D**) Another two examples of fabric reconfiguration that turn the passive fabric butterfly and flower into active ones. Non-stretchable fabric: plain-woven cotton muslin.

In a similar approach, we utilized a single AMF to reconfigure a piece of passive, conventional fabric into an active four-legged structure (Fig. 8A). The routing and programming concept was similar to the one in Fig. 3C. However, we began with a fabric shaped in a four-legged pattern (turtle-like shape, cotton muslin fabric) instead of utilizing a rectangular sheet. As a result, the legs were longer and could raise the structure higher. The structure’s height gradually increased when pressurizing until its legs were perpendicular to the ground. If input pressure kept rising, the legs would bend inward, lowering the structure’s height. The four-legged structure can exert locomotion if its feet are equipped with unidirectional patterns or using multiple AMFs with locomotion operation strategies. Locomotion soft robots are needed in various tasks, including rescue missions from bushfires, collapsed buildings, or hazardous environments, and drug delivery robots for medical applications.

We also leverage the simplicity and versatility of this fabric reconfiguration technique by introducing two other bio-inspired shape-morphing structures (Fig. 8B–D). These shape-morphing structures were reconfigured from passive fabric sheets into active and controllable ones with the help of routing AMFs. Inspired by the Monarch butterfly, we fabricated a butterfly morphing structure using a piece of butterfly-shaped fabric (cotton muslin fabric) and a long AMF stuck under its wings. The wings will bend upward when pressurizing the AMF. Like the Monarch butterfly, both the left and right wings of the butterfly robot were flapping in the same manner because they were controlled by a single AMF. The butterfly was flapping for demonstration only. It could not fly like the Smart Bird (Festo Corp., USA). We also fabricated a fabric flower (Fig. 8D) which had two layers of petals, five petals for each layer. We stuck a single AMF beneath each layer following the outer boundaries of the petals. Initially, the flower was fully blooming, in which all petals were entirely open. Upon pressurization, the AMF caused the bending motion of petals, making them close. Two AMFs controlled the motion of two layers independently while five petals in the same layer were curving at the same time.

Thanks to the fast response characteristic of AMFs, these shape-morphing structures could perform at a comparative speed (2 Hz for the four-legged structure and 1 Hz for the butterfly and the flower) and high durability or repeatability (over 1000 cycles).

## Discussion and conclusion

The textile-based robotic field is undergoing a paradigm shift from using textiles as passive substrates to programmable smart textiles whose compositions consist of stimuli-responsive yarns. Our recently developed soft actuators (or AMFs) which resemble conventional passive yarns in terms of flexibility and high length-per-diameter ratio can elongate back and forth under hydraulic pressure, being excellent candidate elements to fabricate smart and active textiles. We have presented two approaches to creating AMF-based smart textiles: (1) knitting and weaving AMFs; and (2) sticking AMFs to conventional passive fabrics.

In the first approach, we adapted traditional knitting and weaving techniques to construct an AMFs-based knitting sheet, a bidirectional weaving sheet, a unidirectional weaving sheet, and a circular weaving sheet. In their current experimental configurations, these smart textiles could generate an area expansion of 35%, 108%, 65%, and 25%, respectively. Our results are comparable with those in the literature, for example, the knitted textiles made of electroactive polymers achieved less than 5% strain^46^, the active textiles made of McKibben muscles exerted a maximum contraction ratio of 7.1%^45^, the knitted SMA wrist sleeve achieved 36% contraction^42^. Generally, the AMF (which is fluid-driven) has a faster response time compared to thermally-responsive filaments. In addition, the elongation rate of these smart textiles can reach a higher value of at least 250% which is the elongation limit of the AMF as demonstrated in our recent work^49^.

A hysteresis gap occurred in the pressure-elongation hysteresis profiles, being an inherent characteristic of the AMFs. It reflects energy loss when switching from the pressurizing phase to the releasing phase. Experimental data revealed that the hysteresis gaps of knitting and weaving configurations were significantly wider than that of a single AMF^49^. It means that the hysteresis of AMFs is an accumulative attribute. The dead zones found in hysteresis charts represent the initial tension of each particular configuration. Input energy (pressure) is required to overcome these thresholds in order to change the configuration status from static to expanding. The initial tension of AMFs is also an accumulative attribute and is heavily correlated with the elastic properties of the constituent helical coil and silicone tube. When switching the AMF operation mode from pressurizing to releasing, energy loss was in the form of heat. However, since most of our prototypes used very little energy, the energy escape as the heat was unnoticeable. We observed the working of our prototypes under a thermal camera and barely notice any changes in their temperature. The energy usage of our smart textiles was also scalable, meaning that miniature structures will consume even lesser energy so does the escaping heat. We have mathematically developed and experimentally validated analytical models to establish the elongation-force relationship of the knitting and weaving configurations. We were unable to provide an analytical model for bending actuators due to the complexity when incorporating the base material specifications and properties and also the contact status between the AMF and the base.

We also introduced several programmable techniques to create multimodal motion of smart textiles. With suitable constraints, the smart textiles’ ordinary area expansion could be transformed into an s-shape, hyperbolic paraboloid, hollow structure, compression sleeve, bidirectional bending, independently bending and elongating manner. Our smart textile configurations offer versatility, programmability, and shape-shifting capabilities, which are favorable characteristics in the soft robotic field for the development of smart garments, wearable devices, and bio-inspired shape-morphing structures.

In the second approach, we embedded the AMFs into conventional passive fabrics to create desired bending motions and deformations upon pressurization. This simple, fast, and easy method facilitated shape-programmable filaments, whose segments could be mechanically programmed to become active or passive to generate the desired deformations. We also applied this concept to reconfigure passive planar fabric sheets into active and controllable 3D structures. We then demonstrated through several bio-inspired shape-morphing structures such as four-legged structures, a butterfly, and a flower. The ability to transform passive 2D sheets into active 3D structures with desired motions using AMFs opens a new possibility to manipulate objects for use in various domains. For instance, compact and deployable devices for space missions, construction, and industry. It can also be used to create bio-inspired soft robots that provide biomimetic motions for decorations and fashion design as well as wearable assistive devices for haptic display, rehabilitation, and human augmentation.

We have investigated a wide range of shape-shifting capabilities of AMF-based smart textile configurations focusing on their motions (or deformations) rather than their generated force. Although we have introduced different approaches to create different smart textiles for different applications, these works are proof-of-concepts that we believe will provide an alternative technology to the soft robotics-driven smart textiles for many applications, ranging from smart compression garments to shape-shifting robots for search and rescue, or exhibition. Despite advances, several areas need to be improved. Therefore, we suggest that future work should focus on studying comprehensively a particular smart textile configuration in terms of motion, force, and specific applications. Analytical models for bending actuators are desired for control purposes. Another area that could improve the performance of the smart textile is the use of nonlinear hysteresis modeling and adaptive control where real-time output feedback from a soft sensor will be used for closed-loop control^53,54^. Since AMFs can be made meter-long, they can possibly be used as filaments in small-scale knitting, weaving, and embroidering machines to produce smart textiles with desired specifications and high reliability. A study to demonstrate the feasibility of machine-made smart textiles and washable smart textiles is essential to speed up the smart garment industry. For example, our artificial muscle can be used with advanced and automated sewing or embroidery machine such as the ones from Tajima Industries Ltd, Japan to form large-scale smart textile. It is desirable to develop a wearable hydraulic power source to drive smart textiles for further applications. During the experiments, we also noticed that there was a rotational motion of individual AMFs which is due to the nature of the helical coil as the outer constraint layer. However, this rotation slightly affected the elongation of the smart textile. In some configurations where the AMFs were arranged in opposite directions, the rotational effect is unlikely. For some applications where a purely linear extension is highly desired, mathematical models which take into account this effect are highly desired. We observed through demonstrations that the pressure loss was insignificant and the energy conversion was effective even with meter-long AMFs. However, we suggest a comprehensive study on the relationship between pressure loss and AMFs’ specifications to benefit precise control strategies. Finally, further research is required to explore the vast potential of incorporating functional components into smart textiles to provide additional benefits (sensing, variable stiffness) alongside the fundamental actuation.

In conclusion, this article introduces a new class of smart textiles constructed from various configurations of fluid-driven artificial muscles. The proposed smart textiles provide a high degree of versatility and programmability, enabling new possibilities in the soft robotic field, including shape-shifting structures, biomimicking soft robots, locomotion robots, and smart garments. We anticipate this concept will inspire related improvement and development, benefiting the robotic field as a whole.

## Methods

### Material selections and specifications of smart textile prototypes

We used two types of AMFs to configure smart textiles. AMFs type S were made of silicone rubber tube OD 1.19 mm, ID 0.64 mm (Saint-Gobain, France) and stainless steel coil OD 1.49 mm, wire diameter 0.17 mm (Asahi Intecc, Japan). AMFs type M were made of latex rubber tube OD 3.18 mm, ID 1.59 mm (McMaster-Carr, USA) and stainless steel coil OD 3.18 mm, wire diameter 0.33 mm (McMaster-Carr, USA).

The knitting sheet (Fig. 2A) has a dimension of 54 × 52 mm constructed from three L300 mm AMFs type S using the weft knitting technique with three courses and seven wales. The bidirectional weaving sheet (Fig. 2B) has a dimension of 52 × 52 mm constructed from two L400 mm AMFs type S using the plain weaving technique with a pattern of 7 × 7 lines. The unidirectional weaving sheet (Fig. 2C) has a dimension of 57 × 27 mm constructed from one L400 mm AMF type S as warp with seven lines and acrylic yarns as weft. The circular weaving structure (Fig. 2D) has OD 58 mm, ID 18 mm constructed from one L500 mm AMF type M. The three bending actuators (Fig. 3B) have their active-section lengths of 20, 30, and 50 mm constructed from three AMFs type S with the corresponding lengths of 60, 80, and 120 mm. The four-legged structure (Fig. 3C) has a dimension of 60 × 50 mm constructed from one L200 mm AMF type S routing the boundary of a piece of rectangular fabric (cotton muslin fabric). The unidirectional weaving sheet (Fig. 6C) has a dimension of 130 × 80 mm constructed from one L1200 mm AMF type S as warp with nine lines and cotton yarns as weft. The unidirectional weaving sheet (Fig. 6D) has a dimension of 140 × 72 mm constructed from one L920 mm AMF type S as warp with six lines and acrylic yarns as weft.

### Experimental setup for smart textile characterization

The testing platform consisted of a motorized linear slider (Zaber, Canada) that drove a medical syringe (BD Biosciences, Canada) to provide input volume (distilled water) and pressure to a specimen (Supplementary Fig. S1). A pressure sensor (Honeywell, USA) was located right after the syringe outlet to measure input pressure. In the cases of knitting and weaving specimens, their distal ends were connected to a linear slider and an encoder (US Digital, USA) to measure output displacement. A thin elastic string was used to prevent slack when collecting displacement data. The encoder set was replaced with a load cell (Futek, USA) when measuring the contraction force. In the cases of radial expansion and bending actuators, a digital camera was located on top of the platform to capture the specimens’ deformation.

We connected the syringe outlet to all three AMFs of the knitting sheet via a 1-to-3 fluid distributor. In the elongation tests, we drove the syringe plunger to supply hydraulic pressure to the specimen by a 0.1 Hz sinusoidal signal with an amplitude that generated a maximum pressure of 1 MPa. In the force tests, we supplied hydraulic pressure to the specimen until reaching 1 MPa and then connected the specimen’s distal end to the load cell. Subsequently, we withdrew the syringe plunger by a 0.1 Hz sinusoidal signal with an amplitude that kept the minimum hydraulic pressure at 0.1 MPa. We also applied this testing procedure for each AMF of the weaving sheet.

For radial expansion experiments, we gradually increased input pressure to the specimen until reaching a maximum pressure of 1 MPa and then reduced pressure to the initial stage at the same speed. The specimen’s deformation during the testing procedure was recorded by the camera and processed afterward to obtain the area changes. We applied a similar procedure to measure the angle changes of the bending actuators.

## Supplementary Information

Supplementary Video 1.

Supplementary Information 1.

Supplementary Information 2.

## Data Availability

The datasets generated during and/or analysed during the current study are available from the corresponding author on reasonable request.

## References

[1] Gerbode Sharon J, Puzey Joshua R, McCormick Andrew G, Mahadevan L (2012). How the cucumber tendril coils and overwinds. Science.

[2] Forterre Y, Skotheim JM, Dumais J, Mahadevan L (2005). How the Venus flytrap snaps. Nature.

[3] van Manen T, Janbaz S, Zadpoor AA (2018). Programming the shape-shifting of flat soft matter. Mater. Today.

[4] Zhou J, Sheiko SS (2016). Reversible shape-shifting in polymeric materials. J. Polym. Sci. Pol. Phys..

[5] Wang W, Rodrigue H, Ahn S-H (2016). Deployable soft composite structures. Sci. Rep..

[6] Ryu J (2020). Paper robotics: Self-folding, gripping, and locomotion. Adv. Mater. Technol..

[7] Mao G (2020). Soft electromagnetic actuators. Sci. Adv..

[8] Cianchetti M, Laschi C, Menciassi A, Dario P (2018). Biomedical applications of soft robotics. Nat. Rev. Mater..

[9] Calisti M, Picardi G, Laschi C (2017). Fundamentals of soft robot locomotion. J. R. Soc. Interface.

[10] Rogóż M, Zeng H, Xuan C, Wiersma DS, Wasylczyk P (2016). Light-driven soft robot mimics caterpillar locomotion in natural scale. Adv. Opt. Mater..

[11] Lu N, Kim D-H (2013). Flexible and stretchable electronics paving the way for soft robotics. Soft Robot..

[12] Chen Z, Huang G, Trase I, Han X, Mei Y (2016). Mechanical self-assembly of a strain-engineered flexible layer: Wrinkling, rolling, and twisting. Phys. Rev. Appl..

[13] Liu Y, Genzer J, Dickey MD (2016). “2D or not 2D”: Shape-programming polymer sheets. Prog. Polym. Sci..

[14] Janbaz S, Hedayati R, Zadpoor AA (2016). Programming the shape-shifting of flat soft matter: From self-rolling/self-twisting materials to self-folding origami. Mater. Horiz..

[15] Lee H (2019). Directional shape morphing transparent walking soft robot. Soft Robot..

[16] Tolley MT (2014). Self-folding origami: Shape memory composites activated by uniform heating. Smart Mater. Struct..

[17] Mao Y (2015). Sequential self-folding structures by 3D printed digital shape memory polymers. Sci. Rep..

[18] Hawkes E (2010). Programmable matter by folding. Proc. Natl. Acad. Sci. U. S. A..

[19] Siéfert E, Reyssat E, Bico J, Roman B (2019). Bio-inspired pneumatic shape-morphing elastomers. Nat. Mater..

[20] Buckner TL, Kramer-Bottiglio R (2018). Functional fibers for robotic fabrics. Multifunct. Mater..

[21] Sanchez V, Walsh CJ, Wood RJ (2020). Textile technology for soft robotic and autonomous garments. Adv. Funct. Mater..

[22] Quinlivan BT (2017). Assistance magnitude versus metabolic cost reductions for a tethered multiarticular soft exosuit. Sci. Robot..

[23] Chiaradia, D., Xiloyannis, M., Antuvan, C. W., Frisoli, A. & Masia, L. In *2018 IEEE International Conference on Soft Robotics (RoboSoft).* 565–571.

[24] Xiloyannis M, Cappello L, Binh KD, Antuvan CW, Masia L (2017). Preliminary design and control of a soft exosuit for assisting elbow movements and hand grasping in activities of daily living. J. Rehabil. Assist. Technol. Eng..

[25] Hoang TT (2021). A wearable soft fabric sleeve for upper limb augmentation. Sensors.

[26] In H, Kang BB, Sin M, Cho KJ (2015). Exo-Glove A wearable robot for the hand with a soft tendon routing system. IEEE Robot. Autom. Mag..

[27] Hoang TT, Phan PT, Thai MT, Lovell NH, Do TN (2020). Bio-inspired conformable and helical soft fabric gripper with variable stiffness and touch sensing. Adv. Mater. Technol..

[28] Thalman, C. M., Hertzell, T. & Lee, H. In *2020 3rd IEEE International Conference on Soft Robotics (RoboSoft).* 801–807.

[29] Cappello L (2018). Exploiting textile mechanical anisotropy for fabric-based pneumatic actuators. Soft Robot..

[30] Booth JW (2018). OmniSkins: Robotic skins that turn inanimate objects into multifunctional robots. Sci. Robot..

[31] Zhu M, Do TN, Hawkes E, Visell Y (2020). Fluidic fabric muscle sheets for wearable and soft robotics. Soft Robot..

[32] Buckner TL, Bilodeau RA, Kim SY, Kramer-Bottiglio R (2020). Roboticizing fabric by integrating functional fibers. Proc. Natl. Acad. Sci. U. S. A..

[33] Yang B (2021). Reprogrammable soft actuation and shape-shifting via tensile jamming. Sci. Adv..

[34] Shah DS (2020). A soft robot that adapts to environments through shape change. Nat. Mach. Intell..

[35] Sanchez V (2020). Smart thermally actuating textiles. Adv. Mater. Technol..

[36] Hu J, Meng H, Li G, Ibekwe SI (2012). A review of stimuli-responsive polymers for smart textile applications. Smart Mater. Struct..

[37] Wicaksono I, Cherston J, Paradiso JA (2021). Electronic textile gaia: Ubiquitous computational substrates across geometric scales. IEEE Pervasive Comput..

[38] Berzowska, J. & Coelho, M. In *Ninth IEEE International Symposium on Wearable Computers (ISWC'05).* 82–85.

[39] Stylios GK, Wan T (2007). Shape memory training for smart fabrics. Trans. Inst. Meas. Control..

[40] Kim, J. H., Huang, K., White, S., Conroy, M. & Kao, C. H.-L. In *Designing Interactive Systems Conference 2021* 1183–1200 (Association for Computing Machinery, 2021).

[41] Thai MT, Hoang TT, Phan PT, Lovell NH, Do TN (2020). Soft microtubule muscle-driven 3-axis skin-stretch haptic devices. IEEE Access.

[42] Granberry R, Eschen K, Holschuh B, Abel J (2019). Functionally graded knitted actuators with NiTi-based shape memory alloys for topographically self-fitting wearables. Adv. Mater. Technol..

[43] Kurumaya S, Nabae H, Endo G, Suzumori K (2017). Design of thin McKibben muscle and multifilament structure. Sens. Actuator A Phys..

[44] Kurumaya S, Nabae H, Endo G, Suzumori K (2019). Active textile braided in three strands with thin McKibben muscle. Soft Robot..

[45] Hiramitsu, T., Suzumori, K., Nabae, H. & Endo, G. In *2019 2nd IEEE International Conference on Soft Robotics (RoboSoft).* 1–6.

[46] Maziz A (2017). Knitting and weaving artificial muscles. Sci. Adv..

[47] Haines CS (2016). New twist on artificial muscles. Proc. Natl. Acad. Sci. U. S. A..

[48] Haines CS (2014). Artificial muscles from fishing line and sewing thread. Science.

[49] Phan PT, Thai MT, Hoang TT, Lovell NH, Do TN (2020). HFAM: Soft hydraulic filament artificial muscles for flexible robotic applications. IEEE Access.

[50] Phan, P. T. *et al.* Twisting and braiding fluid-driven soft artificial muscle fibers for robotic applications. *Soft Robot. *(2021). 10.1089/soro.2021.0040 34613831

[51] Phan PT (2021). Smart surgical sutures using soft artificial muscles. Sci. Rep..

[52] Xiong J, Chen J, Lee PS (2021). Functional fibers and fabrics for soft robotics, wearables, and human–robot interface. Adv. Mater..

[53] Thai MT (2021). Design, fabrication, and hysteresis modeling of soft microtubule artificial muscle (SMAM) for medical applications. IEEE Robot. Autom. Lett..

[54] Hassani V, Tjahjowidodo T, Do TN (2014). A survey on hysteresis modeling, identification and control. Mech. Syst. Signal Process..

