# Supplementary material for: Smart textiles using fluid-driven artificial muscle fibers
Source: Sci Rep. 2022 Jun 30;12:11067. doi: 10.1038/s41598-022-15369-2 (PMC9247081; doi:10.1038/s41598-022-15369-2)
Supplement: Supplementary file 2 — Supplementary Information 1. [file 41598_2022_15369_MOESM2_ESM.docx]

**Video Tittle**: Performance of smart textiles

**Legend**: This video introduces the performance of the smart textile and its applications.
