# Supplementary material for: Smart textiles using fluid-driven artificial muscle fibers
Source: Sci Rep. 2022 Jun 30;12:11067. doi: 10.1038/s41598-022-15369-2 (PMC9247081; doi:10.1038/s41598-022-15369-2)
Supplement: Supplementary file 3 — Supplementary Information 2. [file 41598_2022_15369_MOESM3_ESM.docx]

Supporting Information

**Smart Textiles Using Fluid-Driven Artificial Muscle Fibers**

**Phuoc Thien Phan^1^, Mai Thanh Thai^1^, Trung Thien Hoang^1^, James Davies^1^, Chi Cong Nguyen^1^, Hoang-Phuong Phan^2^, Nigel H. Lovell^1^, and Thanh Nho Do^1,^***

^1^ Graduate School of Biomedical Engineering, Faculty of Engineering, University of New South Wales (UNSW), Sydney, NSW 2052, Australia

^2^ School of Mechanical and Manufacturing Engineering, Faculty of Engineering, University of New South Wales (UNSW), Sydney, NSW 2052, Australia

* Corresponding author, e-mail: [tn.do@unsw.edu.au](mailto:tn.do@unsw.edu.au)

1. **Experimental setup for smart textile characterization**


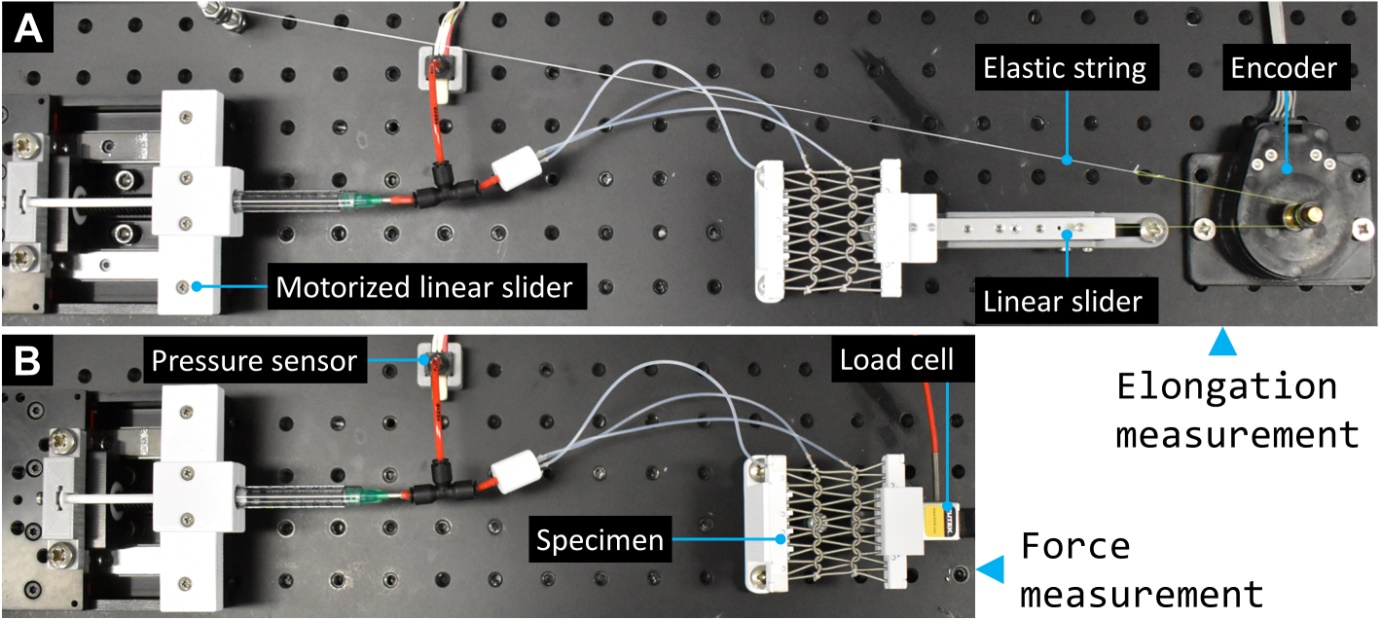


**Figure S1.** Experimental setup to measure output elongation (A) and force (B) of specimens when receiving hydraulic pressure.

1. **Characteristics of smart textiles regarding input volume**

Since all specimens were made of artificial muscle fibers (AMFs), the overall trend of experimental results (Fig. S2) agrees with the AMF’s fundamental characteristic where the input volume has a proportional relationship with output elongation and a reverse proportional relationship with contraction force.

Each AMF of the weaving sheet received 0.2 mL input fluid volume to generate approximately 30% elongation (Fig. S2A). The volume-elongation hysteresis chart showed a narrow gap between the pressurizing and releasing phases, indicating a good response of AMFs to hydraulic in both expansion and contraction motions. (Fig. S2A). The weaving sheet could exert a contraction force of 5.6 N after releasing input volume and pressure (Fig. S2B). The volume-force hysteresis chart also showed a small gap between the releasing curve and the pressurizing curve. The weaving sheet’s area expansion relies on the volume amplitudes supplied to each of the two AMFs, which are shown in the three-dimensional surface plot (Fig. S2C). Experiments also revealed that the weaving sheet could generate an area expansion of 66% when its warp and weft AMFs simultaneously received 0.2 mL fluid volume.

Experimental results of the knitting sheet have a similar pattern to that of the weaving sheet, including the narrow hysteresis gap in the volume-elongation chart. Interestingly, the releasing curve overlapped the pressurizing curve in the volume-force chart. The knitting sheet generated 30% elongation when receiving 0.43 mL fluid volume and subsequently exerted a contraction force of 9 N (Fig. S2D and S2E).

In the case of the circular weaving sheet, there was an expansion from its initial area to 25% after receiving 0.7 mL fluid volume (Fig. S2F). There was a dead zone of input volume until 0.14 mL before the specimen started to expand. This dead zone is created by the initial tension of the AMF. Fig. S2F also showed that the releasing curves almost overlapped the pressurizing curves, denoting inconsiderable energy loss when switching the circular sheet motions.

Experimental results for three bending actuators (fabric reconfiguration) showed that their hysteresis profiles share similar patterns (Fig. S2G), in which they underwent a short dead zone before rising. We supplied the same volume of fluid (0.035 mL) to the three bending actuators (L20, L30, and L50 mm). However, each actuator generated different bending angles. The L20 and L30 mm actuators reached a bending angle of 167° and 194°, respectively. The longest bending actuator (L50 mm) achieved the largest bending angle of 236°. The volume-angle hysteresis chart also showed a relatively narrow gap between the pressurizing and releasing curves of all three bending actuators.


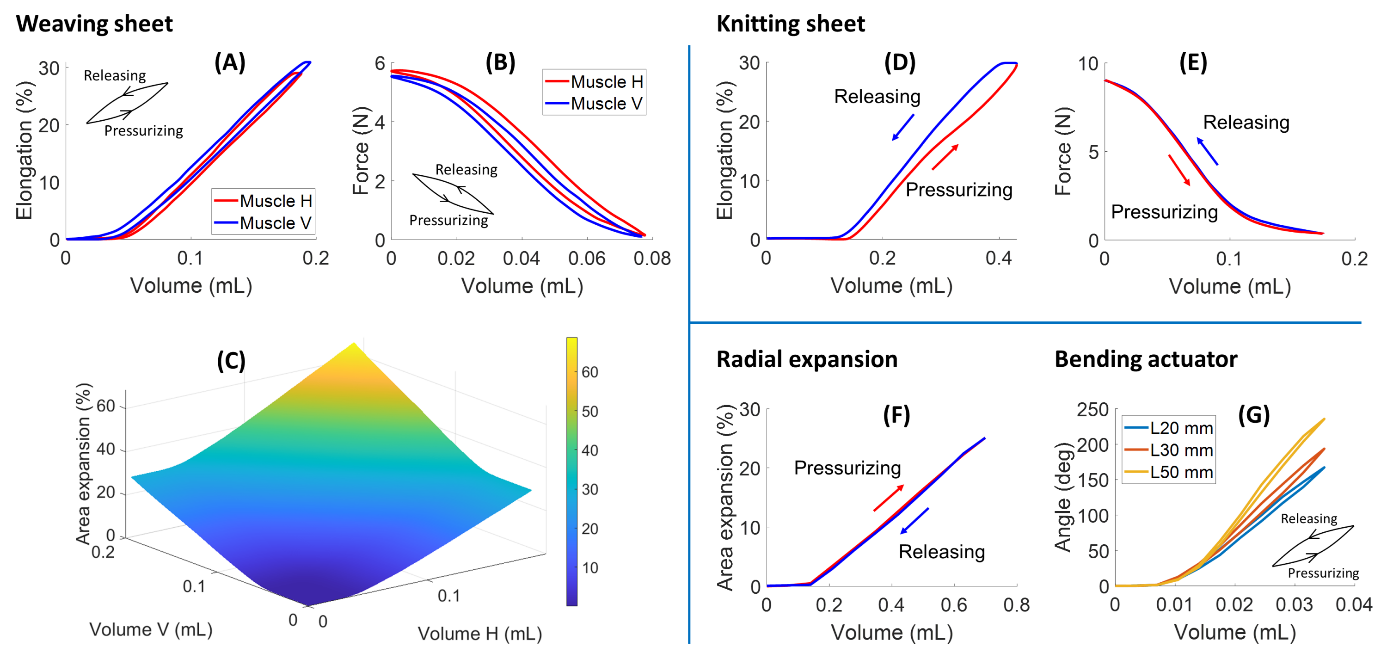


**Figure S2.** Characteristics of smart textile configurations. (A, B) Hysteresis profiles of input volume and output elongation and force of the weaving sheet. (C) Area expansion of the weaving sheet. (D, E) Relationship between input volume and output elongation and force of the knitting sheet. (F) Area expansion of the radial expansion structure. (G) Bending angles of three different lengths of bending actuators.

1. **Analytical models to establish the elongation-force relationship**
   1. **Knitting sheet**

Considering a knitting sheet made of *n* similar AMFs (or *n* courses), each has an outer diameter *d* and forms *m_k_* loops (or wales) (Fig. S3A). Initially, the knitting sheet had a dimension of *h_ki_* × *v_ki_* in horizontal and vertical directions, respectively. It was assumed that the AMFs were uniformly knitted, resulting in analogous loops. Each typical loop was identified by the width *g_i_* (*g_i_* = *h_ki_* /*m_k_*), length *u_i_*, and looping angle *ϕ_i_* at the initial phase. There were overlaps between two adjacent courses, whose width was approximately *g_i_*/2. Relationships between these initial parameters are described as follows:

| $v_{ki}=nu_{i}-(n-1)\frac{g_{i}}{2}$ |  |
| --- | --- |
| $\Rightarrow u_{i}=\frac{2v_{ki}+(n-1)g_{i}}{2n}$ | (1) |
| $\varphi_{i}=\tan^{-1} \frac{g_{i}}{2\left( u_{i}-g_{i} \right)}$ | (2) |

When applying pressure *P_k_* to all AMFs, the knitting sheet elongated by *ε_kv_* in the wale direction and reached a new length *v_kp_* = *v_ki_* (1 + *ε_kv_*). Subsequently, it exerted a contraction force *F_kv_* when connecting to an external load and releasing input pressure. We observed that the knitting sheet was stretched in the wale direction and shrunk in the course direction simultaneously when working against an external load. *μ* is the ratio between the course strain (*ε_kh_*) and the wale strain (*ε_kv_*), *μ* = *ε_kh_* /*ε_kv_*, and is called the Poisson’s ratio of the knitting sheet. Experiments revealed *μ* was closely related to the knitting loop aspect ratio, *μ* ≈ *g_i_* /*u_i_*.

At the stretching phase, the knitting sheet had a new dimension of *h_kp_* × *v_kp_*, where *h_kp_* = *h_ki_* (1 – *με_kv_*) and *v_kp_* = *v_ki_* (1 + *ε_kv_*). Also, each knitting loop had a new width *g_p_* (*g_p_* = *h_kp_* /*m_k_*), length *u_p_*, and looping angle *ϕ_p_*.

| $u_{p}=\frac{2v_{kp}+(n-1)g_{p}}{2n}$ | (3) |
| --- | --- |
| $\varphi_{p}=\tan^{-1} \frac{g_{p}}{2\left( u_{p}-g_{p} \right)}$ | (4) |

Each loop consisted of two legs (Fig. S3A), whose length increased from *l_ki_* at the initial phase to *l_kp_* for the stretching phase, where *l_ki_* = (*u_i_* – *g_i_*)/cos*ϕ_i_* and *l_kp_* = (*u_p_* – *g_p_*)/cos*ϕ_p_*. *x_kv_* is the displacement of each leg, namely *x_kv_* = *l_kp_* – *l_ki_*.

The maximum contraction force *F_out_* of an AMF (when input pressure *P* = 0) was given in reference,^1^ and is reintroduced as follows:

| $F_{out}=\alpha EA_{0}\left( 1-\frac{1}{1+x/{l_{i}}} \right)+kx$ | (5) |
| --- | --- |

where *α*, *E*, *A_0_* represents the stretch ratio, Young’s modulus, and cross-sectional area of the silicone tube, respectively; *k* is the stiffness coefficient of the helical coil; *x* and *l_i_* are the displacement and initial length of the AMF, respectively.

The last constituent *kx* in Eq. (5) describes the helical coil elastic force *F_c_* which is assumed to be linear-elastic or Hookean. However, the accumulative initial tension of helical coils in knitting and weaving configurations must be considered. Therefore, we propose a comprehensive equation to describe the coil force, as shown in Eq. (6). The coil force equation consists of a short linear segment at the first 2% strain followed by a modified Hookean segment that accounts for the initial tension.

| $F_{c}(x)=\left\{ \begin{aligned} qx, \varepsilon\leq2\% \\ kx+F_{0}, \varepsilon>2\% \end{aligned} \right.$  Continuity condition: $q=k+{F_{0}}/x, \varepsilon=2\%$ | (6) |
| --- | --- |

where *ε* and *F_0_* are the strain and initial tension of the helical coil, respectively.

Since the knitting sheet works against an external load in the wale direction, its contraction force is equivalent to that of a single course. There are 2*m_k_* legs for each course. The contraction force of each leg follows Eq. (5), thus the knitting sheet contraction force can be expressed as:

| $F_{kv}=2m_{k}\left[ \alpha EA_{0}\left( 1-\frac{1}{1+{x_{kv}}/{l_{ki}}} \right)+\left\{ \begin{aligned} qx_{kv}, \varepsilon_{kv}\leq2\% \\ kx_{kv}+F_{0}, \varepsilon_{kv}>2\% \end{aligned} \right. \right]\cos\varphi_{p}$  Continuity condition: $q=k+{F_{0}}/{x_{kv}}, \varepsilon_{kv}=2\%$ | (7) |
| --- | --- |

- 1. **Weaving sheet**

Consider a weaving sheet made of two AMFs (H and V, each has an outer diameter *d*) that follows a *m_h_* × *m_v_* lines criss-cross pattern (Fig. S3B). The weaving sheet dimension is *h_wi_* × *v_wi_* at the initial stage. It is assumed that the distance between two adjacent lines is identical for each AMF. Thus, the distance between two lines of the AMF H is *s_vi_* = *v_wi_* /(*m_h_* – 1) and the distance between two lines of the AMF V is *s_hi_* = *h_wi_* /(*m_v_* – 1). Since two AMFs are similar, we solely present here the equation derivation for the AMF H. In plain weave, when a line crosses over or under another line, it forms an angle with the transverse orthogonal plane. We refer to this angle as an interlocking angle, *θ_hi_* for lines of the AMF H.

| $\theta_{hi}=\tan^{-1} \frac{d}{s_{hi}}=\tan^{-1} \frac{d(m_{v}-1)}{h_{wi}}$ | (8) |
| --- | --- |

The AMF H has *m_h_* lines with the same length *l_hi_* = *h_wi_* /cos*θ_hi_*. When applying pressure *P_h_* to the AMF H, the weaving sheet elongates *ε_wh_* horizontally and reaches a new length *h_wp_* = *h_wi_* (1 + *ε_wh_*) and a new interlocking angle *θ_hp_*.

| $\theta_{hp}=\tan^{-1} \frac{d(m_{v}-1)}{h_{wp}}=\tan^{-1} \frac{d(m_{v}-1)}{h_{wi}\left( 1+\varepsilon_{wh} \right)}$ | (9) |
| --- | --- |

The new length of weaving lines is *l_hp_* = *h_wp_* /cos*θ_hp_*. Let *x_wh_* be the displacement of a line, given as *x_wh_* = *l_hp_* – *l_hi_*. Adapting to Eq. (7), the weaving sheet contraction force in direction H can be expressed as:

| $F_{wh}=m_{h}\left[ \alpha EA_{0}\left( 1-\frac{1}{1+{x_{wh}}/{l_{hi}}} \right)+\left\{ \begin{aligned} qx_{wh}, \varepsilon_{wh}\leq2\% \\ kx_{wh}+F_{0}, \varepsilon_{wh}>2\% \end{aligned} \right. \right]\cos\theta_{hp}$  Continuity condition: $q=k+{F_{0}}/{x_{wh}}, \varepsilon_{wh}=2\%$ | (10) |
| --- | --- |


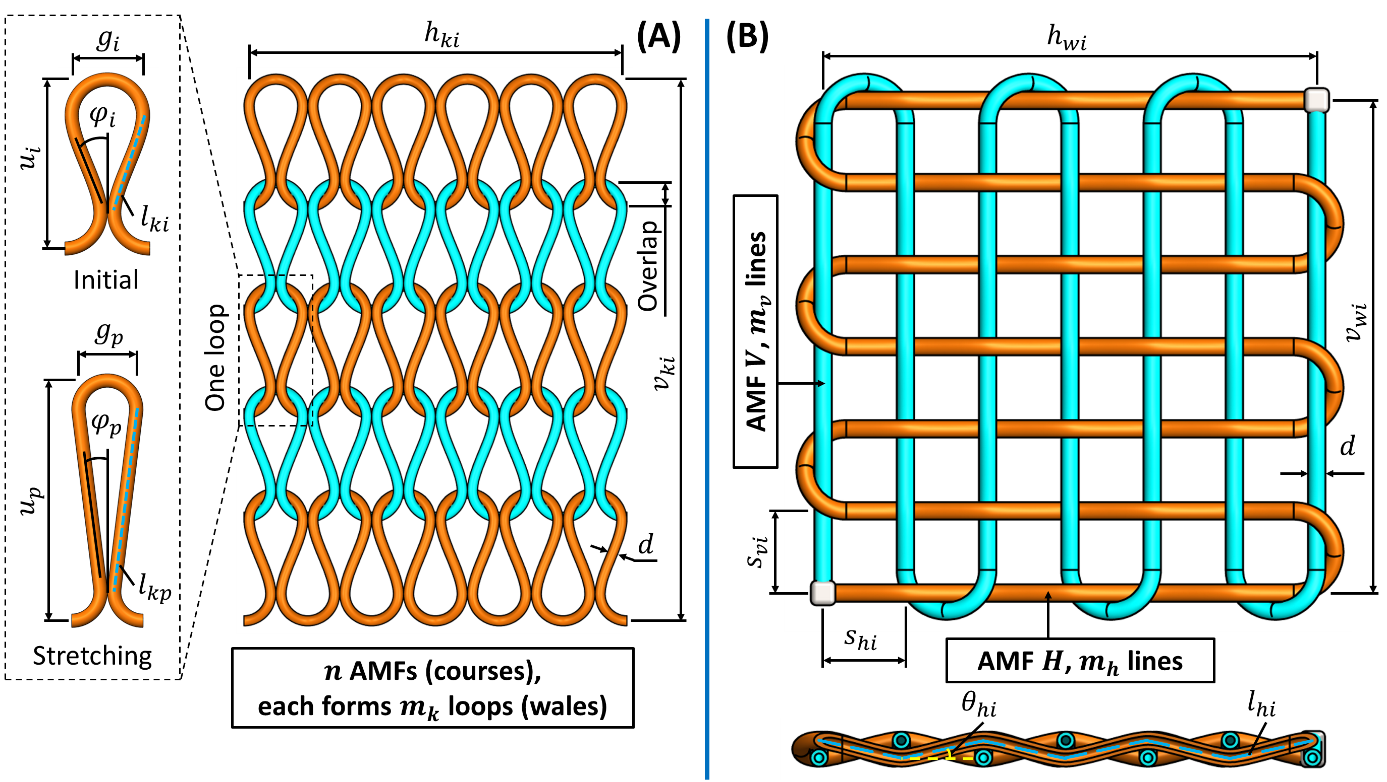


**Figure S3.** Analytical model illustration to establish the elongation-force relationship. (A) Knitting sheet. (B) Weaving sheet.

1. **Shape-programmable filaments**

We have developed a simple technique to create a bending actuator – by attaching an AMF to a strip of non-stretchable fabric. This section extends the bending actuator concept to shape-programmable filaments, in which we can strategically allocate multiple active and passive segments in a single AMF to produce the desired shape.

We first attached an AMF to a strip of non-stretchable fabric by a layer of double-sided tape, creating a single active filament. Later, we deliberately added another layer of tape at locations that we intended to be passive (Fig. S4A). Note that multiple strips of fabric can be attached to one AMF. Also, the length and direction of these strips vitally affect bending curvature and direction of the active segments. Fig. S4B and S4C demonstrate four active filaments have been programmed to transform their shapes from straight lines into letters (UNSW) upon pressurization. This simple technique enables the shape-shifting capability of AMFs to turn 1D lines into 2D shapes and possibly 3D structures.


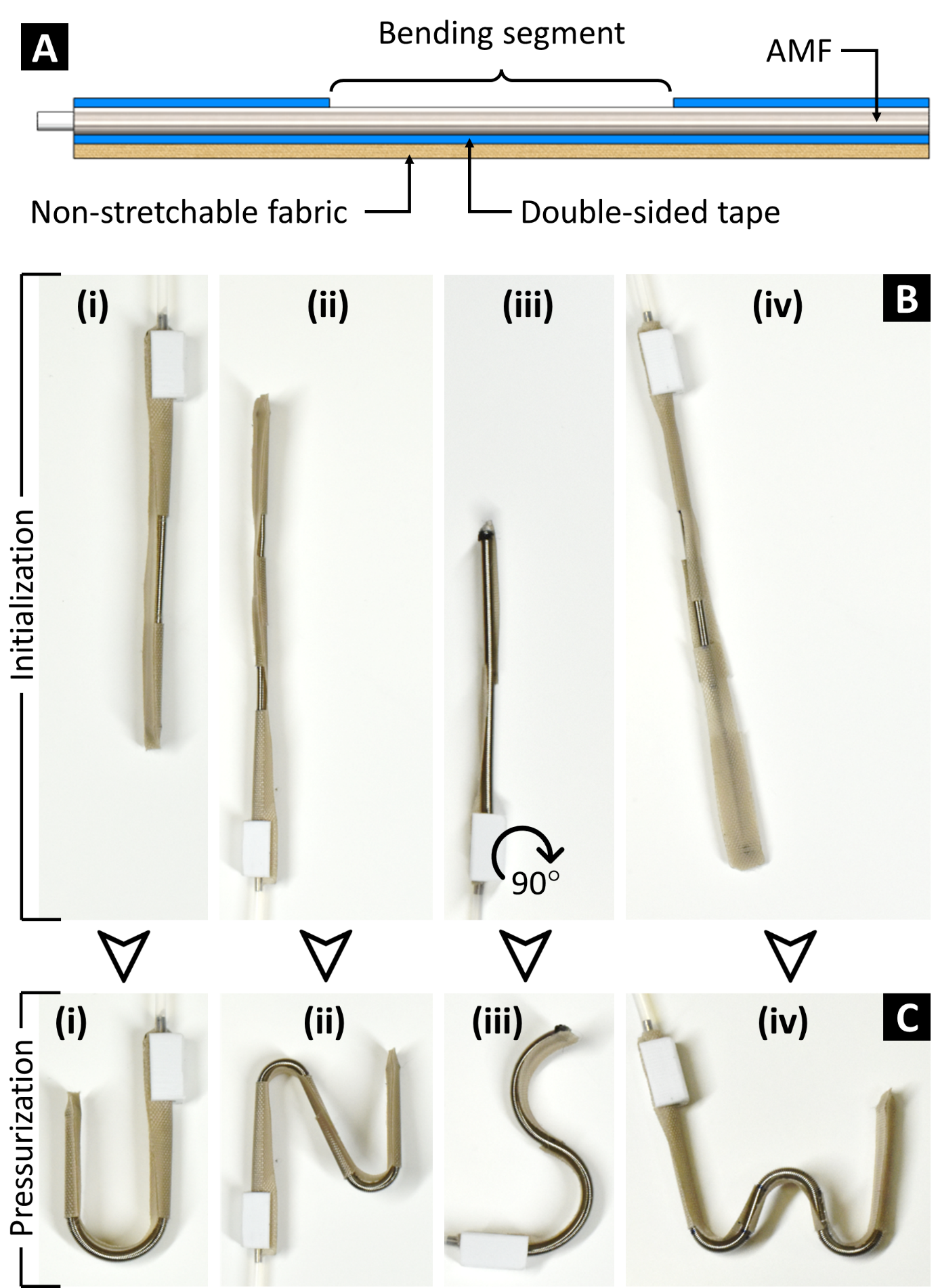


**Figure S4.** Shape-programmable filaments. (A) Attaching an AMF to a strip of non-stretchable fabric to create a bending actuator. (B, C) Programming four bending actuators to form four letters (UNSW) upon pressurization.

**References**

1 Phan, P. T., Thai, M. T., Hoang, T. T., Lovell, N. H. & Do, T. N. HFAM: soft hydraulic filament artificial muscles for flexible robotic applications. *IEEE Access*. **8**, 226637-226652. <https://doi.org/10.1109/ACCESS.2020.3046163> (2020).
